# A Survey on Low-Latency DNN-Based Speech Enhancement

**DOI:** 10.3390/s23031380

**Published:** 2023-01-26

**Authors:** Szymon Drgas

**Affiliations:** Institute of Automatic Control and Robotics, Poznan University of Technology, Piotrowo 3A Street, 60-965 Poznan, Poland; szymon.drgas@put.poznan.pl

**Keywords:** low-latency speech enhancement, DNN, computational cost

## Abstract

This paper presents recent advances in low-latency, single-channel, deep neural network-based speech enhancement systems. The sources of latency and their acceptable values in different applications are described. This is followed by an analysis of the constraints imposed on neural network architectures. Specifically, the causal units used in deep neural networks are presented and discussed in the context of their properties, such as the number of parameters, the receptive field, and computational complexity. This is followed by a discussion of techniques used to reduce the computational complexity and memory requirements of the neural networks used in this task. Finally, the techniques used by the winners of the latest speech enhancement challenges (DNS, Clarity) are shown and compared.

## 1. Introduction

The goal of the speech enhancement task is to process a noisy speech input signal and provide an estimate of clean speech. The performance of such systems can be measured in terms of intelligibility and quality of the estimated clean signal (for example, using objective metrics such as spectro-temporal objective intelligibility (STOI) [1] or perceptual evaluation of speech quality (PESQ) [2]). Speech enhancement can be applied to mobile phones or hearing aids. Some applications need low-latency processing; i.e., the delay between the estimated clean signal in relation to the noisy signal cannot be too big. Otherwise, the application of such a system will not result in an improvement in speech communication. Additionally, in real-world applications, speech enhancement algorithms can be constrained by the capabilities of mobile hardware. Another important aspect is energy consumption. Even if the hardware is capable of guaranteeing a certain latency, energy consumption can be an issue.

Recently, deep neural networks (DNNs) turned out to be an effective means for the speech enhancement task for non-stationary additive noise. There are two important groups of DNN-based speech enhancement systems: the spectral domain and the time domain. The systems that work in the spectral domain process a noisy speech spectrogram, and the results are in the form of a mask that is used to multiply the noisy speech spectrogram to obtain an estimate of clean speech. There are also systems that directly estimate the clean speech signal spectrogram instead of a mask. In the case of time-domain signals, both the input and output signals are in the time-domain form (for example, [3]).

In the group of spectral-domain speech enhancement systems, there are systems in which the magnitude of the spectrogram of the noisy speech is processed with a neural network to predict the magnitude spectrum of the clean signal or by using masks which, after entry-wise multiplication of the noisy spectra, estimate the magnitude spectrum of the clean signal. Commonly used masks are ideal binary masks (IBM) or ideal ratio masks (IRM) [4]. The output signal from the enhancement system is reconstructed by combining the estimated magnitude spectrum of the clean signal and the phase of the noisy speech input. There is also a mask which is applied to the magnitude spectrogram, but it takes into account the difference between the phase of the clean and noisy speech signals. This mask, known as a phase-sensitive mask (PSM), was proposed in [5]. The newer solution is to input the complex spectrum of noisy speech on the input of a neural network trained to predict the complex spectrum of clean speech or the complex ideal ratio mask (for example, [6]. The complex spectrogram is often represented as a three-dimensional tensor in which one of the dimensions is equal to two: one index value for the real and the other for the imaginary part of a complex number. Such a complex spectrum can be processed using convolutional layers and treat the real/imaginary part’s dimension as a feature dimension. There are also implementations in which real convolutional layers are used to implement complex multiplication addition operations.

In the first DNN-based speech enhancement systems, a frame or several concatenated spectrogram frames were provided at the input of the DNNs. The larger context generally results in a larger number of parameters and poor generalization of the networks [7,8]. Convolutional neural networks such as [9] can achieve greater receptive fields without significantly increasing the number of parameters. In this case, the input spectrogram is processed by convolutional layers, and the receptive field can be increased by adding more layers and reducing the spectro-temporal resolution. Convolutional layers used in neural networks for speech enhancement often use a dilation rate greater than one, helping to exploit long-range dependencies without increasing the number of model parameters. In [10], dilated convolutions were applied in a neural network processing magnitude spectrogram. Another speech enhancement system with dilated convolutions was proposed in [11] and tested with reverberated data. In [12], the dilated convolutions were applied for input and output signals in the time domain. Other types of neural network layers used in speech enhancement are recurrent, such as long-short-term memory (LSTM) or gated recurrent units (GRUs). They have been shown to effectively incorporate context information about the speaker [13]. There are also combinations of convolutional and recurrent layers as in [14]. The recurrent layer was applied to extend the receptive field in the U-net architecture (described in Section 3.2) [15]. This idea was extended by using recurrences or dilated convolutions at each level, as in [16,17]. Recently, multi-head attention [18] has been used for speech enhancement for time-domain inputs and outputs [19]. Self-attention was also tested within the U-net architecture [20] and with convolutional layers [21]. This technique provides an alternative way to take into account potentially large contexts of the input signal. Neural models that use self-attention are often called transformer networks. There is much interest in self-attention recently; rather, much research is being devoted to optimizing ways to encode positional information in sequences in the input to transformer networks. The absolute positional encoding from the original research paper [18] was extended, and relative [22] and continuous dynamic [23] positional encodings have also been proposed, but their performance in speech enhancement has not yet been tested. Many of the deep neural networks presented in the literature are not causal; that is, they use the future signal to estimate the current part of the signal. Examples of using non-causal layers include using future frames in networks with fully connected layers in [24] or the application of non-causal convolutions and bidirectional recurrences in [14]. To obtain low-latency speech enhancement, causal variants of the convolutional and self-attention layers should be used.

The speech enhancement methods mentioned above are summarized in Table 1. For each work in the above paragraphs, there are indicated inputs and outputs to the neural network. In order to give an idea of the neural network architecture proposed in a given work, the types of layers are mentioned. Additionally, information on datasets and the size of training, validation, and test subsets is mentioned as well. The speech datasets that were used in the mentioned works are TIMIT [25], WSJ0 [26], Voice Bank [27], and Libri speech [28]. The datasets that contain noises and environmental sound are DEMAND [29], Noisex [30], FreeField [31], and Auditec CD (available at http://www.auditec.com, accessed on 22 January 2023).

Additionally, the latency of DNN-based speech enhancement can be enforced by the way in which feature extraction is performed. For example, in systems based on short-time Fourier transform (STFT) or mel-frequency cepstral coefficients (MFCCs), the signal is divided into short segments; this is performed by multiplying the signal with a window of certain length and stride. Latency is related to the length of the window. Even in DNN-based speech enhancement that does not use feature extraction, the latency can come from the size of the windows.

In addition to the latency related to the kind of feature extraction and DNN architecture, an important aspect is the computational time. Many successful DNN architectures require much more computational operations and memory than would actually be needed to achieve a certain level of performance (often measured in terms of metrics such as STOI or PESQ). This results from the fact that an overparameterized deep neural network (DNN) provides a simplified optimization landscape, which ensures local optimal points that are close to the global one [32]. There are techniques that can be used to reduce the computational and memory requirements of neural networks. They include neural network compression methods such as pruning, quantization, and tensor decomposition.

The objective of this paper is to analyze the existing literature on low-latency speech enhancement. More specifically, the objective is to compare the properties of causal DNN layers that have proven to be effective for the speech enhancement task and provide cues when a given unit should be used. Next, I describe the spectrogram calculation techniques for low-latency speech enhancement. This is followed by a discussion about techniques for the compression of the models. Afterwards, a summary of the architectural elements that are used in the systems that performed best in DNS and clarity challenges is provided. The three main questions in this survey are as follows:

1. What are the properties of various causal DNN units, and how do they perform in speech enhancement systems?
2. How can we compress models based on what we know about various techniques from the literature? How does compression work in speech enhancement?
3. What techniques are used in speech enhancement systems that have won speech enhancement challenges?

The paper is structured as follows. In Section 2, the general processing scheme in the low-latency speech enhancement system is presented, along with their sources of latency and tolerable values. In Section 3, causal elements that can be used to build a neural network for speech enhancement are discussed. In Section 4, techniques for the compression of the DNNs are presented. The winning systems in the DNS and Clarity challenges are described in Section 5. Finally, the conclusions are presented in Section 6.

## 2. Real-Time Processing of Signals Using DNNs

**Figure 1:**
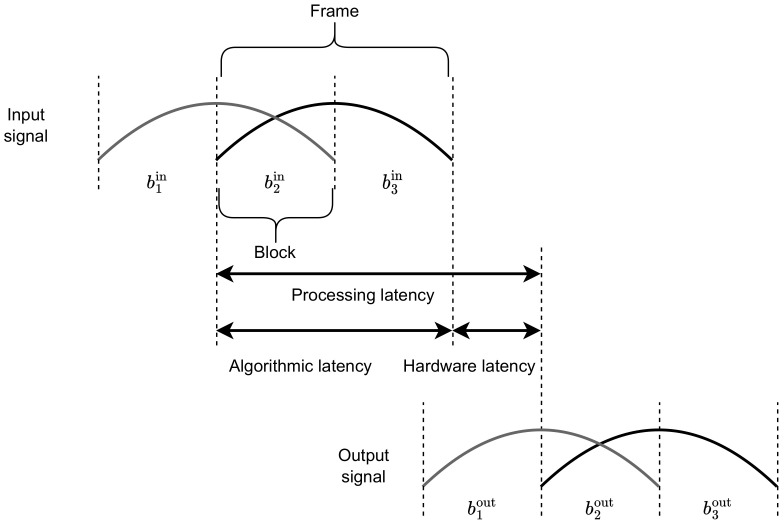
Illustration of processing in frames, where $b_{1}^{\mathrm{in}},b_{2}^{\mathrm{in}},b_{3}^{\mathrm{in}}$ are the input blocks, while $b_{1}^{\mathrm{out}},b_{2}^{\mathrm{out}},b_{3}^{\mathrm{out}}$ are the output blocks.

Real-time processing aims to modify a signal and output the result with an acceptably low latency. The input signal is processed by the hardware in blocks $b_{1}^{\mathrm{in}},b_{2}^{\mathrm{in}},\ldots$, each of size $n_{b}$. Each time $n_{b}$ samples are collected, they are processed by the speech enhancement system, and the resulting block $b_{n}^{\mathrm{out}}$ is provided to the output. In many systems, however, the signal is processed in frames with some overlap (i.e., the stride of the frame is lower than its length). An example is shown in Figure 1. In this case, the stride is the same as the length of the block, while the frame length is $2n_{b}$. The processed signal block $b_{2}^{\mathrm{out}}$ can be provided in the output after processing the entire frame (which spans $b_{2}^{\mathrm{in}}$ and $b_{3}^{\mathrm{in}}$). This part of latency is called algorithmic latency. Next, hardware latency is the time in which the frame is processed by the hardware. The algorithmic and hardware latencies are added up to determine the processing latency, which is related to the delay between the input and output of the speech enhancement system.

The latency that is tolerable in audio communication is 150 ms [33]. For listeners with normal hearing, the tolerable asynchrony between mouth and speech is 200 ms (speech delayed to video). For users of cochlear implants, the acceptable asynchrony is 200–250 ms [34]. The tolerance to delay is lower for people who use hearing aids. The reason comes from the fact that, in addition to auditory–visual synchrony, both speech perception and speech production must be considered. When all these various factors are taken into account, delay tolerances of only 20 to 30 ms are obtained [35,36,37].

### Inputs and Outputs, State

In this subsection, I consider general schemes in DNN-based causal speech enhancement systems. The goal of DNN-based speech enhancement systems is to transform a noisy signal x(n) for n=1,…,N, where *N* is a number of samples into a signal y(n) with better intelligibility and quality. A direct case is where the *n*’th output sample is computed from all input samples collected so far:

(1) y(n)=f(x(1),…,x(n)).

where f() is a function representing a neural network. This formulation is impractical as the input to the neural network grows from sample to sample. In many cases, the current history input sample and $T_{s}-1$ previous input samples are provided at the input

(2) $$y\left(n\right)=f(x\left(n-T_{s}+1\right),\ldots ,x\left(n\right))\;.$$

Such speech enhancement systems may have an algorithmic latency of one sample. However, they need to propagate through the network for every sample, which may cause a large hardware latency. In practice, signals are processed in blocks, and to compute output samples for a given block, the input samples of the block and the $T_{s}-1$ input samples from the preceding blocks must be provided at the input of the DNN. The example of a network that uses formulation (2) is WaveNET [38], which uses dilated convolutions to obtain a high receptive field without drastically increasing the number of parameters of the speech enhancement network.

More often, the signal is processed using frames. That is, the input signal is windowed with a window of a certain length and hop-size. A neural network for speech enhancement accepts at its input the current input frame and $T_{f}-1$ previous input frames. At its output, the current frame of the clean signal is predicted. The neural network can be formulated as:

(3) $$y_{k}=f\left(x_{k-T_{f}+1},\ldots ,x_{k}\right)\;,$$

where $y_{k}$ is the current output vector, while $x_{k}$ is the *k*’th input vector. In the case of such a formulation, the algorithmic latency is the size of one frame.

An example of a neural network that accepts raw signal framed in its input is [39]. Conv-TasNet [40] can also be considered within this formulation, as it starts with a convolutional layer with significant stride. The drawbacks of time-domain processing by neural networks are a poor generalization ability and the fact that speech and noise are more separable in the time-frequency domain [41]. They also give worse results in terms of MOS in DNS challenges.

In many popular networks, the vectors x contain the result of the fast Fourier transform (FFT) of the windowed inputs. In some networks, it is a magnitude spectrum, and in others, it is a complex spectrum, which can be provided as two real numbers for each frequency. There are also works (such as [42]), in which features such as MFCCs, AMS, etc., are provided at the input.

From the perspective of outputs, there are two main groups of speech enhancement systems that process vectors (as in Equation (3)). In one of them, the raw signal, or FFTs of the input signal, is predicted. There are also methods in which a chosen mask is predicted [43]. The mask is used to multiply the spectrum element-wise. The rationale for predicting masks rather than a spectrum is that the dynamics of the outputs can be limited, and hence the training of the neural network is easier.

In the above considerations, there are networks that accept at their input a signal with some history. This scheme is correct for networks with causal, fully connected, convolutional, or transformer units. There is also a possibility of using recurrent units. In this case, the vectors that characterize the state for the previous samples should also be taken into account. This can be formulated as

(4) $$\left(y_{k},h_{k}^{1},\ldots ,h_{k}^{H}\right)=f\left(x_{k-T_{f}+1},\ldots ,x_{k},h_{k-1}^{1},\ldots ,h_{k-1}^{H}\right)\;.$$

Each time neural network inference is performed, the output vector and the *H* vectors that broadly characterize the signal processed so far are obtained. They are computed from the current local signal and the state vectors from the previous time step.

## 3. Elements of Causal Neural Network Architectures

### 3.1. Recurrent Layers

**Figure 2:**
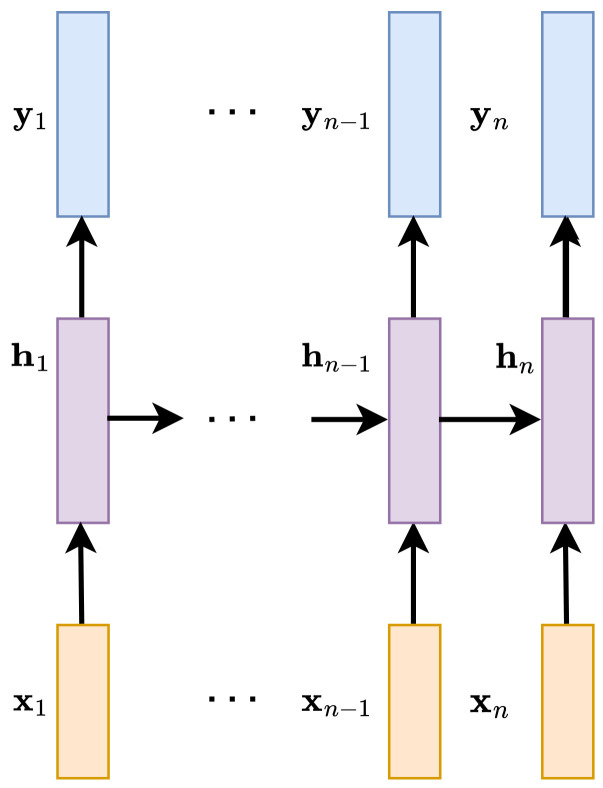
Schematic illustration of the dependence between input vectors ($x_{1},\ldots ,x_{n}$), state vectors ($h_{1},\ldots ,h_{n}$), and output vectors ($y_{1},\ldots ,y_{n}$) in the RNN layer.

Recurrent neural networks can be used to exploit the context of the input signal in speech enhancement. In the case of temporal recurrences, only recurrences in the advancing time direction can be used. The inputs to the layer are feature vectors, for example, frames of the input spectrogram. The schematic dependence between the input, state, and output vectors is shown in Figure 2. At each time step *n*, to compute the current state $h_{n}$, the state vectors from the previous step (for example, $h_{n-1}$) and the current input have to be provided, $x_{n}$. There are several types of recurrent units used in speech enhancement, such as long short-term memory (LSTM) [44,45] or gated recurrent units (GRU) [46]. Although recurrent units may summarize a very long history, studies compare them with dilated convolutions, showing that this is actually not the case [47].

Another issue connected to recurrent neural networks is the large number of parameters when they are used in a simple setup, that is, when the input frame of dimension of hundreds of components is transformed to the state using a fully connected layer. There is also a solution to this problem in which each frequency band is processed by an independent recurrent unit, and the recurrent units of all frequencies have shared parameters [16,48].

The performance of RNN, specifically LSTM for speech enhancement, was demonstrated in [13]. It was shown that LSTMs without access to future spectrogram frames performed better in terms of STOI than DNNs with access to future frames. To reduce the number of parameters in the neural network that utilize RNNs, the sequence of feature vectors for each frequency along the time axis can be modeled independently.

### 3.2. Convolutional Layers

In speech enhancement, convolutional layers have proven to be very efficient. They can be applied to both spectral and raw-waveform audio representations. It is common to use 1D and 2D convolutional layers. When 2D convolutional layers are used, for real-time speech enhancement, causality needs to be ensured only on the time axis. Convolutional layers can be easily transformed to its causal variants by defining masks only for non-positive indices in the time axis; that is, the current output depends only on the previous and current inputs. The size of the context of input on which the current output depends is called a receptive field. The size of the receptive field increases as the number of convolutional layers increases. It can be even bigger, without increasing the number of network’s parameters, by using strided or dilated convolutions.

In the case of a convolutional layer, in which the stride in the time dimension is larger than one, the temporal resolution becomes smaller, which can result in a reduction in the number of computations and the memory required to store the resulting activations. However, before reconstruction, upsampling has to be performed, typically with the transposed convolutional layer. The transposed convolution for the stride *k* can be described as an insertion of k−1 zeros between subsequent input activations and processing with a convolutional layer (possibly causal). It should be emphasized that in most of the implementations, the zeros are not inserted between the activations, but the convolutions take into account proper activations from the preceding layer.

**Figure 3:**
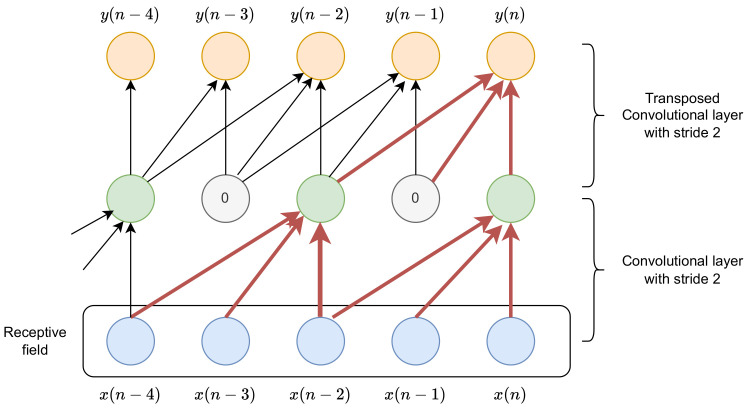
A schematic illustration of dependence in time axis for the composition of convolutional layer with a stride equal to 2 and a transposed convolution with a stride of 2.

Figure 3 presents a scheme showing the dependence of activations on inputs for the two-layer convolutional network. Note that the input can be samples of raw waveform or vectors representing spectrogram frames. The scheme is also valid in the context of 2D convolutions, but it is visualized as the receptive field only in the time dimension. A similar scheme can be obtained independently for the frequency dimension. In the network illustrated in Figure 3, the first layer is a convolutional layer with a causal kernel of a size of 3 and a stride of 2. Hidden activations are obtained every second input. Every hidden activation depends on the input from n−2 to *n*. Next, each output depends on hidden activations from *n* to n−2, but the activations for time steps for which they were not calculated have to be treated as zeros. Thus, in the depicted case, the receptive field spans inputs for five time steps. However, the receptive field for y(n−1) spans inputs from only three time steps (from x(n−4) to x(n−2)). This can be avoided when the kernel size is the integer multiplicity of the stride.

**Figure 4:**
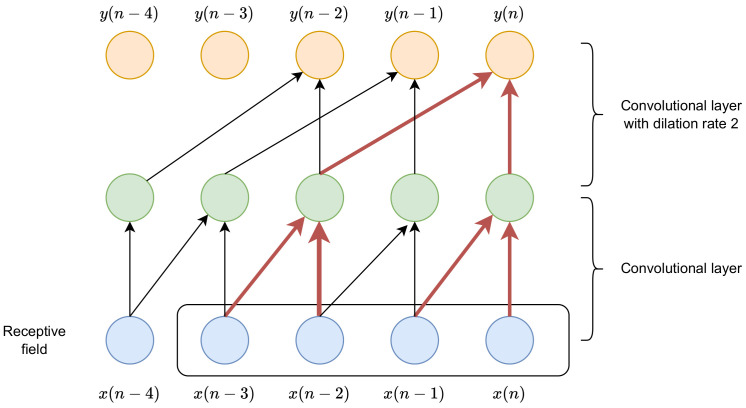
A schematic illustration of time dependence for a composition of two layers convolutional and convolutional with dilation rate of 2.

Another technique used to enlarge the receptive field in the time dimension is to use dilated convolutional layers. In the kernels of dilated convolutional layers [49], only every *d*’th sample is nonzero, where *d* is called the dilation rate. An illustration of the use of dilated convolutional layers is presented in Figure 4. In the first layer, the dilation is set to one (ordinary convolution), and the kernel size is two. In the second layer, the kernel size is also two, but the dilation rate is set to two. It can be noticed that the size of the receptive field is four, while the composition of two normal convolutions (with a dilation rate equal to one) would result in a receptive field size of three.

The causal convolutional layers can be used as the main building blocks in the U-net architecture, which is popular in speech enhancement. The U-net architecture was originally used in the medical image segmentation task [50]. Its main parts are the encoder and the decoder. In the encoder, there are several levels. It is possible that on each level, the encoder, together with the convolutions, downsamples the signal’s representation, for example, using strided deconvolution. The decoder is typically the mirror reflection of the encoder; i.e., at each level, together with convolution, the representation is upsampled, for example, using transposed convolutions. The corresponding levels of the encoder and decoder are connected by skip connections, which may prevent the loss of spatial information that may result from downsampling. The examples of using U-net-based architectures for speech enhancement are [51,52].

An example of using causal convolutional layers for speech enhancement is given in [53]. A neural network was applied to the spectrogram frames, together with the unidirectional recurrent layer (specifically LSTM, which is described in Section 3.1). The combination of convolutional layers and LSTMs gave better performance in terms of STOI and PESQ than the LSTM-only network.

Another example is TasNet (time-domain audio separation network), a neural network that can be applied to speech signals in the time domain, in which STFT is replaced with a convolutional layer with a sufficient stride. For each position of the convolutional kernel, non-negative features are computed. To perform the separation, the features are multiplied by a mask computed from the features with LSTM layers. Later, another version of Tas-NET was proposed, called Conv-TasNet, in which the LSTM layers in the separator were replaced by dilated convolutions. The shared idea in TasNet and Conv-TasNet is to replace the STFT step for feature extraction with a data-driven representation that is jointly optimized with an end-to-end training paradigm. The idea used in Conv-TasNet is based on the temporal convolutional network (TCN) proposed in [47] as a replacement for RNNs. The TCNs are made up of temporal convolutional modules (TCMs). Each TCM has three blocks with dilation; a dilation block is formed by stacking residual blocks having exponentially increasing dilation rates (1, 2, 4, 8, 16, 32, etc.). Each residual block has (1,1) convolution and depthwise convolution and a (1,1) convolution, PReLU, and batch normalization.

### 3.3. Self-Attention

**Figure 5:**
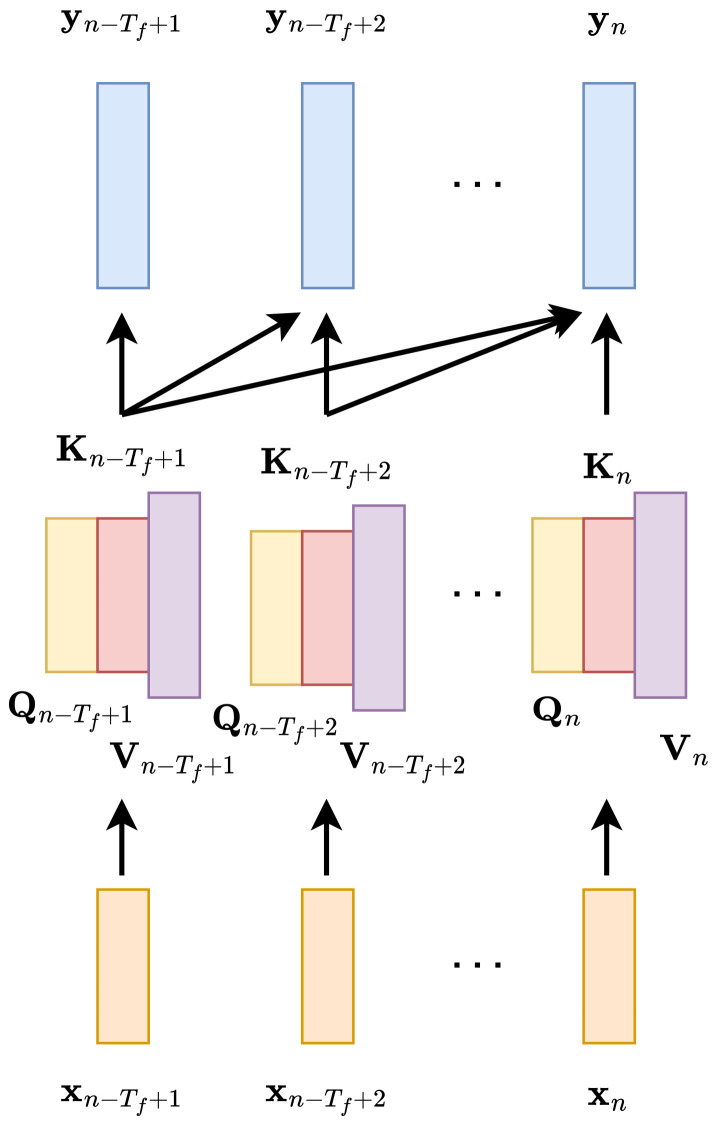
A graph representing dependence of output vectors $y_{n-T_{f}+1},\ldots ,y_{n}$ on vectors computed in the self-attention layer: keys $K_{n-T_{f}+1},\ldots ,K_{n}$, queries $Q_{n-T_{f}+1},\ldots ,Q_{n}$, and values $V_{n-T_{f}+1},\ldots ,V_{n}$. The vectors in the self-attention layer are computed from the input vectors $x_{n-T_{f}+1},\ldots ,x_{n}$.

Self-attention [18] is a mechanism that allows the context to be parameterized. Its architecture is depicted in Figure 5. Given a sequence of $T_{f}$ input vectors $x_{n-T_{f}}+1,\ldots ,x_{n}$, self-attention computes the output sequence of the output vectors $y_{n-T_{f}+1},\ldots ,y_{n}$, and all these vectors depend on all input vectors. From each input vector, three vectors are obtained using a linear map: query $q_{n}$, key $k_{n}$, and value $v_{n}$. In the case of local self-attention [54,55], the context is limited and the vector $q_{n}$ is compared to $k_{n-T_{f}+1},\ldots ,k_{n}$. The comparison is made by means of the scaled dot product and transformed by the sigmoid function as

(5) $$s\left(q,k\right)=\mathrm{sigmoid}\left(\frac{k^{T}q}{\sqrt{n}}\right)\;.$$

Scores *s* are used to weight the aggregation of vectors $v_{n}$ as

(6) $$y_{n}=\sum_{n^{\prime }=n-T_{f}+1}^{n}s\left(q_{n},k_{n^{\prime }}\right)v_{n}\;.$$

Although the vectors are obtained by linear transformation from x, Pandey et al. [39] reported that according to their experiments, linear transformation is not necessary.

The number of parameters does not grow together with the size of the receptive field. The disadvantage of self-attention is that the number of computations grows with the size of the receptive field, both during training and inference.

During inference, we need to compare the vector $q_{n}$ with all keys in the context. The bigger the context, the more comparisons that have to be computed.

During training, it is often not necessary to restrict the history to frames $T_{f}$, as training is typically performed using *T* frame excerpts from a signal. Then, all comparisons between queries and keys can be stored in the T×T matrix. To guarantee the causality of the system, we can use a mask defined as

(7) $$M_{nn^{\prime }}=\left\{\begin{array}{cc} 1 & n\leq n^{\prime }\\ -\infty & n>n^{\prime } \end{array}\right.\;.$$

Thus, the scores quantifying the similarity between the query and the key are computed as

(8) $$s\left(q_{n},k_{n^{\prime }}\right)=\mathrm{sigmoid}\left(M_{nn^{\prime }}+\frac{k_{n^{\prime }}^{T}q_{n}}{\sqrt{n}}\right)$$

For each frame, many different output vectors $y_{n}^{1},\ldots ,y^{H}$ can be obtained by extracting queries, keys, and values using layers with differently initialized trainable parameters. In this case, different aspects of the context are parameterized. This is known as multi-head attention.

In [56], multi-head attention blocks were used to build the speech enhancement network. At the input, the network accepts the magnitude spectrogram of the noisy speech. Next, trigonometric positional encoding is added, and following that, MHA blocks consisting of MHA and fully convolutional blocks are cascaded. This network, named MHANet, was compared with ResLSTM [57] and ResNET [58]. MHANet was able to produce better results in terms of quality and intelligibility than those obtained by RNN and TCM. This is because MHA is better able to model the long-term dependencies of noisy speech than RNNs and TCMs. Furthermore, it was found that no positional encoding for the attention mechanism is required for speech enhancement to surpass the mentioned baselines (ResLSTM and ResNET).

In [59], speech enhancement was described using local self-attention. The results obtained in this work suggest that increasing the window of local self-attention over one second does not result in better performance in terms of STOI, ESTOI, and PESQ.

### 3.4. Comparison of the Properties of the Causal Layers

The differences between various aspects of causal layers are summarized in Table 2. The types of layers are compared by receptive field, computational aspects of training and inference, and the complexity of the implementation. The comparison is related to the case in which a sequence of *N* input *D*-dimensional vectors is provided at the input of a recurrent or self-attention layer or the sequence (stack) of convolutional layers. The output is a sequence of output *N* vectors of dimension *H*.

#### 3.4.1. Receptive Field

In the case of recurrent layers, the receptive field does not directly depend on the number of trainable parameters. Although the recurrent architecture potentially allows for the dependence of the output on an infinite context, there are experimental results that show that this is not the case [47]. The receptive field in convolutional layers depends on the size of the kernel. The stack of convolutional layers (a sequence of layers) can additionally enlarge the receptive field, especially when strided or dilated convolutions are used (see Section 3). To obtain the receptive field that spans *N* input vectors, $\log_{K}N$ dilated convolutional layers are needed. In the case of local self-attention, the receptive field depends on the size of the window that spans the input vectors whose keys are compared to the query from the current time step.

#### 3.4.2. Computational Complexity

The comparison of the complexity of different layers of a neural network can be found in [60,61]. In the case of the recurrent layer (GRU or LSTM), the computational complexity is $O(NHD+NH^{2})$. In the case of LSTMs both terms are calculated four times, whereas for GRUs, they are calculated three times. The computational complexity of the recurrent layers depends quadratically on *H*. Therefore, the complexity depends mainly on the dimensionality of the hidden state. In the case of a convolutional layer, the complexity is O(NDKH), that is, it depends linearly on the dimensionality of the input and hidden vectors, as well as on the size of the kernel. The self-attention layer has complexity $O(ND\left(H+H_{1}\right)+N^{2}\left(H_{1}+H\right))$. Thus, it depends quadratically on the length of the input sequence of vectors. Note that in the case of causal layers, only the lower/upper triangular part of N×N matrix with query-key comparisons must be computed. It should also be mentioned that, in order to better characterize the context, more heads are used. Thus, the number of computational operations must be multiplied by the number of heads.

#### 3.4.3. Training

In the case of recurrent layers, during training, unfolding is performed; that is, a computational graph is constructed, with the copy of a recurrent unit for each time step. It can result in a very large computational graph and, therefore, high memory and speed requirements. Thus, recurrent layers can be very expensive for training, especially in the case of high temporal resolution, when the number of time steps in each example is large. In [62], this combination of recurrent layers with multi-layer perceptron was shown reduce the computational cost. Another important characteristic is the maximum length between the input and output vectors in the neural network [18]. For example, it can be the number of computational elements between the first input vector and the *N*’th output vector. In the case of recurrent layers, it is O(N), and thus it depends linearly on the length of the sequences. In the case of dilated convolutions, it is $O(\log_{K}N)$, while in the case of self-attention, it does not depend on *N*. Another important difference between the recurrent layers and the others is that the number of sequential operations depends on *N*. Hence, the possibilities of parallelization of computations during training are limited.

#### 3.4.4. Inference

When a speech enhancement system works in blocks or frames (see Section 2), inference using a neural network is performed when a block/frame is provided. In the case of recurrent layers, along with the input, the hidden vector from the previous block/frame should be given as well. Each time, the complexity of the inference depends quadratically on *H*. In the case of a convolutional layer, together with the current input, the previous N−1 inputs must also be provided. As mentioned in the previous subsections, the number of layers with dilations to obtain the receptive field that spans *N* input vectors is $\log_{K}N$. It is the case that many of the intermediary activations between input and output were already computed in for the previous block/frame. However, this is difficult to avoid using standard components from libraries such as TFLite or ONNX runtime. Thus, efficient implementation, which avoids this redundancy, can be complex. An example of a non-redundant implementation for convolutional layers can be found in [63]. In the case of attentional layers, the query for a given time step is compared to the previous keys. In online systems, it is reasonable to compare the current query with keys from a specified number of previous keys. On the basis of these comparisons, the feature vector is computed for the current frame. Thus, the number of feature vectors that can affect current features is bounded by the window size.

### 3.5. Time/Spectral

Many speech enhancement systems use a noisy speech spectrogram as input to the neural network, which outputs the spectrogram of the clean speech estimate. The spectrogram is built from FFTs of the signal in the window. Algorithmic latency is determined by the size of the window in which a speech signal is processed. However, reducing the length of the window results in a lower-frequency resolution if FFT is performed. In [64], an asymmetric scheme was proposed to tackle this problem. The processing scheme is based on an *L*-sample Hann window. The window pair is defined of lengths *L* and 2M, where L>2M. The reduction in speech enhancement performance when the window length is reduced was discussed in [65]. The windows are configured so that their product is equal to the prototype window of 2M. The effectiveness of such asymmetric STFT is evaluated in [66]. Similarly, asymmetric windowing based on square root Hann, rectangular, and Tukey was reported in [65].

## 4. Techniques for the Reduction in Computational and Memory Requirements of DNNs

Although a DNN architecture for speech enhancement can be designed from the beginning to obtain low computational and memory requirements (for example, MobileNets [67]), there are techniques in which a well-performing deep neural network (in terms of quality or intelligibility) can be transformed into a neural network that needs less computations and memory during inference. The most common techniques are pruning, quantization, factorization, knowledge distillation, and skip-RNN, which are described in the following subsections.

### 4.1. Pruning

In many cases, pruning deep neural networks is a three-stage process. The first stage is training a possibly large neural network. Next, some of its weights are selected according to a certain criterion (for example, the absolute value of the weight) and set to zero. Finally, the pruned neural network can be fine-tuned.

The selection of weights that should be set to zero is typically carried out according to a criterion chosen in a way that will not significantly affect the performance of the neural network. The criterion is based on some measure of its importance/significance in achieving the desired performance of the initial network. In [68], it is mentioned that most pruning methods are divided into two groups: in one group, the performance change (reflecting quality or intelligibility) is measured after setting a weight to zero is measured. In the other group, terms are added to the objective function, which reward the network for more effective solutions (with many weights with values close to zero). For example, a term proportional to the sum of all weight absolute values favors solutions with small weights; those that are nearly zero are not likely to influence the output much and thus can be eliminated. In [69], the criteria for choosing the weights of which the error is not sensitive are divided into data-agnostic and data-driven. In the category of data-agnostic methods, we can enumerate the following:

- The criterion based on Hessian;
- Merging weights by value similarity [70];
- Pruning weights with L2 norms below a given threshold [24,71].

Data-driven pruning techniques are:

- Average percentage of zeros of neurons;
- Entropy of activations to remove channels in the CNN;
- Remove activations with a flat gradient.

The above-mentioned pruning techniques can reduce the number of operations and memory requirements for a given neural network. However, it does not have to speed up the inference. This happens because the weights are replaced by sparse matrices. In order to make pruning effective in terms of speech, the pruning of structural components (for example: whole neuron, convolutional filter, etc.) has to be employed. The effectiveness of structural pruning can be enhanced by using additional terms in the objective function. However, this time, there is a need for group sparsity terms which force the weights of all structural elements to be zero.

In the context of speech enhancement, pruning was used in [72,73]. In [72], several speech enhancement architectures were pruned: a network with three fully connected layers, a recurrent network with four hidden LSTM layers, a temporal convolutional neural network, and a gated convolutional neural network.

Pruning is conducted for each weight tensor individually by gradually reducing the number of preserved weights. After each reduction, the change in the loss function is calculated for the validation dataset. If the change in the loss function exceeds the specified threshold value, the process is stopped, and the network is fine-tuned. To perform structured pruning, group sparsity is used, where, in the case of convolutional layers, each kernel is treated as a weight group for pruning for both recurrent and fully connected layers; in weight matrices, the columns are treated as weight groups. The pruning process is similar to that of unstructured pruning, but instead of removing the prespecified percentage of weights, the weight groups are pruned according to their l1-norm. To increase the efficiency of pruning, the loss terms that impose sparsity are added.

The experimental results reported in [72] suggest that by pruning, the number of weights can be significantly reduced. The performance of the resulting models was compared to that of small models with a number of parameters comparable to those of the pruned models. It was observed that the larger networks after pruning give better results than the small networks trained from scratch. The authors also carried out an experiment to test whether per-tensor pruning is more efficient than pruning with common threshold. It turned out that the per-tensor method gives higher PESQ and STOI. The authors found that training and pruning an over-parameterized DNN achieves better enhancement results than directly training a small DNN that has a size comparable to the pruned DNN.

In [74], a pruning method was evaluated using a deep denoising autoencoder neural network. The experimental results showed that the iterative pruning method with retraining could remove 50% of the network parameters without affecting the network performance in subjective speech perception tests.

### 4.2. Quantization

In many deep neural networks, the single-precision floating-point format [75] is used to represent both weights and activations. This format comes mainly from the fact that it is a default representation of numbers on GPUs. In this subsection, quantization techniques are described which result mainly in reduction in the model’s size, where instead of storing each weight using a 32-bit number, a look-up table with centroids can be stored. The reduction in the size of the model is also important in the context of the throughput required to read the weights from memory. Quantization of weights and activations can also reduce the required computations.

The reduction in the size of the speech separation model was considered in [76]. The authors proposed a low-bit quantization method based on nonuniform and dynamic quantization methods (where the parameters of the quantization are adjusted according to the data). The method was evaluated on a speech enhancement task, to which a two-layer neural network with fully connected layers was used. The results show that the size of the model (in bytes) can be reduced by 50% with a reduction in STOI of 2.7% in terms of STOI compared to the case without quantization.

In the technique described above, the quantization is applied to the weights of an already trained network. To avoid reduction in the performance of the quantized neural network, quantization-aware training [77,78] can be employed. In this method, during training, quantization (using a low-precision representation of numbers) is simulated during the forward pass, while the backward pass remains the same. This results in taking into account the quantization error in the loss function. This can reduce performance degradation caused by quantization.

In [72], the weights of the pruned network are quantized using VQ. The weights of each tensor are partitioned into *C* clusters. Once the VQ converges, the weights are reset with the value corresponding to the centroid. Each weight can be represented as a cluster index. During inference, the value of each weight is looked up in the codebook. To choose a suitable value, the number of centroids is gradually increased, and the value of the loss function on the validation set is monitored. Experiments with DNN with fully connected layers, recurrent neural networks, and temporal convolutional neural networks (TCNN) [12] show that the quantization method substantially reduces the size of the model without degrading the enhancement performance. For example, for the LSTM model, the size of the model was reduced five times, while the differences for STOI and PESQ are 0.002 and 0.01, respectively.

Approaches that quantize only the weights are primarily concerned with storage on the device and less with computational efficiency [79]. On the other hand, networks such as binary, ternary, and bit-shift are concerned with computational efficiency. However, these methods provide computational benefits on custom hardware, but not on existing hardware. The authors of [79] proposed a quantization scheme to quantize both weights and activations to 8-bit integers and just a few trainable parameters, such as 32-bit integers (bias vectors). The method can be applied on existing integer-only hardware. The results reported by the authors suggest that the method improves the trade-off between accuracy and latency.

It is also possible to speed up the inference of a neural network with appropriately quantized weights by replacing multiplications by additions [80]. It is assumed that the activations and parameters are in single floating point format. The property exploited in this method is that, when one of the operands has a mantissa value equal to zero, the floating-point multiplication can be replaced by the integer addition (with the additional use of some bias). The proposed method was tested for the speech enhancement task using a fully convolutional neural network. In the experimental results, the inference time was reduced by up to 20%. Compared to the original FCN models, IA-Net improved the inference time by 1.19× to 1.21× and compressed the model size by 71.9%. However, with such significant acceleration and compression, the quality and intelligibility scores were even better in terms of PESQ.

### 4.3. Tensor Decomposition

In neural networks, linear operations (matrix multiplication/convolution) are interleaved with nonlinear operations, which are usually entry-wise (for example, ReLU nonlinearity which does not have any parameters or PRELU with two parameters). Most of the trainable parameters of neural networks are in arrays that specify linear operations. For example, the fully connected layer is specified by the weight matrix, and convolutional filters are in the form of fourth-order tensors. These parameters can be redundant, as pointed out in [81,82]. To avoid redundancy, the matrices and tensors can be factorized into a product of smaller tensors. This can result in lower memory requirements. Moreover, a linear operation is replaced by a sequence of linear operations that in total require a smaller number of arithmetic operations than the original single linear operation. In the literature, there are works that demonstrate the efficiency of tensor decomposition methods in neural networks. The application of tensor decomposition results in a change in the architecture of neural networks (the mentioned replacement of one linear operation into a sequence of smaller operations of smaller cost). The modified network is then randomly initialized and trained from scratch. There is also a possibility to use the weights of the pre-trained network and factorize it into the values of smaller tensors using, for example, higher-order singular-value decomposition (HOSVD) [83]. In [84], the above-mentioned techniques were applied to well-known architectures (ResNet [85], VGG [86]) for image classification. These architectures contain both convolutional and fully connected layers. In the case of fully connected layers, truncated singular-value decomposition was used to factorize their weight matrices while tensors representing convolutional layers were factorized using Tucker-2 [87] and nested Tucker-2 decompositions [84]. The results obtained for the image classification task for MNIST [88] and CIFAR-10 [89] showed a small decrease in accuracy while reducing both the memory requirement and computational complexity.

There are also techniques in which a matrix is reshaped into a high-order tensor, which is known as tensorization. Next, the high-order tensor is factorized using a chosen tensor decomposition technique. This results in a reduction in memory requirements and, sometimes, computational complexity. Let us start with the I×J matrix; it can be reshaped into a tensor of dimensions $I_{1}\times \ldots \times I_{N}$, where $I=\prod_{i=1}^{N/2}I_{i}$ and $J=\prod_{i=N/2+1}^{N}I_{i}$. Next, it can be factorized using, for example, a tensor train method in which the tensor of order *N* is factorized into a product of tensors as

$$G_{i_{1},\ldots ,i_{N}}=\sum_{j_{1}=1}^{R_{1}}\ldots \sum_{j_{N}=1}^{R_{N}}U_{i_{1},j_{1}}^{\left(1\right)}U_{j_{1},i_{2},j_{2}}^{\left(2\right)}\ldots U_{j_{N-1},i_{N-1},j_{N}}^{\left(N-1\right)}U_{j_{N},i_{N}}^{\left(N\right)}\;,$$

for $i_{l}=1,\ldots ,I_{l}$ and l=1,…,N. For example, a weight matrix of size 1000×1000 from the fully connected matrix can be reshaped into tensor 10×10×10×10×10×10 obtained from tensors $U^{\left(1\right)},\ldots ,U^{\left(6\right)}$. If $R_{1}=\ldots =R_{6}=3$, then $U^{\left(1\right)}\in R^{10\times 3}$, $U^{\left(2\right)}\ldots U^{\left(5\right)}\in R^{3\times 10\times 3}$, and $U^{\left(6\right)}\in R^{3\times 10}$. Therefore, instead of 1 million parameters, it is needed to store only 30+4·90+30=420.

There is also similar decomposition known as a matrix product operator (MPO), in which an I×J matrix is reshaped into an $I_{1},\ldots ,I_{N},J_{1},\ldots ,J_{N}$ tensor where $I=\prod_{i=1}^{N}I_{i}$ and $J=\prod_{i=1}^{N}J_{i}$. Then, the tensor is decomposed in the following

$${\bar{G}}_{i_{1},\ldots ,i_{N},j_{1},\ldots ,j_{N}}=\sum_{k_{1}=1}^{R_{1}}\ldots \sum_{k_{N}=1}^{R_{N}}{\bar{U}}_{i_{1},j_{1},k_{1}}^{\left(1\right)}{\bar{U}}_{k_{1},i_{2},j_{2},k_{2}}^{\left(2\right)}\ldots {\bar{U}}_{k_{N-1},i_{N-1},j_{N-1},k_{N}}^{\left(N-1\right)}{\bar{U}}_{k_{N},i_{N},j_{N}}^{\left(N\right)}\;,$$

where ${\bar{U}}^{\left(1\right)}$ is of size $I_{1}\times J_{1}\times R_{1}$, tensors ${\bar{U}}^{\left(i\right)}$ are $R_{i-1}\times I_{i}\times J_{i}\times R_{i}$ for i=2,…,N−1, and ${\bar{U}}^{N}$ is of size $R_{N-1}\times I_{N}\times J_{N}$. Thus, the number of entries in the resulting model can be computed as

$$I_{1}J_{1}R_{1}+\sum_{i=2}^{N-1}I_{i}J_{i}R_{i-1}R_{i}+R_{N-1}I_{N}J_{N}\;.$$

Matrix product operators were applied to neural networks for speech enhancement in [90]. The weight matrices in linear transformations in fully connected and LSTM layers were replaced by matrix product operators. The experimental results show that the compressed method outperformed pruning in terms of STOI. It was also found that computational complexity decreased with compression rate. The MPO decomposition format can be carefully selected to maintain or reduce the computation complexity. The MPO-based model compression method can be integrated to any network models with the linear transformations.

In [91], various tensor decomposition were tested in sequential modeling tasks using a polyphonic music dataset. It was observed that tensor train decomposition performed better than Tucker decomposition with a similar number of parameters. CANDECOMP/PARAFAC (CP) [92] decomposition performed better than Tucker, with a similar but slightly worse performance than tensor train.

In [93], tensor train decomposition was applied to reduce the number of parameters without significant loss of performance. In contrast to earlier works on the use of tensor train decomposition to compress a neural network, Qi et al. proposed using a technique for choosing a proper rank. Furthermore, their method was evaluated on, among others, speech enhancement, in which it resulted in better STOI and the same PESQ while reducing the size of parameters from 30 Mb to 0.5 Mb.

### 4.4. Knowledge Distillation

Knowledge distillation (KD) [94], known also as teacher–student training, refers to training small DNN models by supervisions generated by computationally demanding teacher models. Low-cost E3Net [95] also use KD to leverage unpaired noisy samples. E3Net outperformed earlier networks proposed by authors with a three times reduction in computational cost. Additionally, knowledge distillation schemes resulted in further compression of the model (2–4 times with a slight degradation in quality metrics).

Kobayashi et al. [96] used knowledge distillation to train a neural network with unidirectional recurrent layers via the supervision of a network with bidirectional layers. The knowledge distillation technique applied to a causal network resulted in better performance in terms of better naturalness and perceptual speech intelligiblity in noisy conditions.

### 4.5. Skip-RNN

Although RNN-based neural networks can potentially model very long dependencies for speech enhancement, there are problems connected to them during training, such as vanishing gradients and difficulties in capturing long-term dependencies. In [97], Skip-RNN was proposed. The idea is to extend a recurrent unit with updates. Fewer updates translate into fewer required sequential operations to process an input signal, faster inference, and lower energy consumption. The skip-RNN can be considered as time dynamic pruning.

In [98], skip-RNN was applied in a speech enhancement neural network. The network processes spectrograms with a number of LSTM layers followed by fully connected layers. It was shown that after pruning and quantization, the system satisfied the requirements (memory and latency) of the used hardware (STM32F746VE MCU). After application of the skip-RNN technique, the number of operations was reduced 1.78 times, at the cost of higher memory needs, but still satisfying the constraints imposed by the hardware. It is also mentioned that, although the speech enhancement performance metrics behave well after the application of the skip-RNN, skip-RNN increases the memory of the network and its ability to model long-term dependencies, even for gated units. During training, gradients are propagated through fewer updating time steps, providing faster convergence in some tasks involving long sequences. The skip-RNN strategy was applied to a speech enhancement task in which a spectrogram was processed by a neural network with encoder–decoder architecture, with DPRNN layers between encoder and decoder. In [99], the skip-RNN strategy is proposed, in which the state of the RNNs is updated intermittently and, therefore, a large amount.

### 4.6. Comparison of the Properties of the Compression Methods for the Reduction in Computational and Memory Requirements

The techniques for the reduction in computational and memory requirements described in Section 4.1, Section 4.2, Section 4.3, Section 4.4, Section 4.5 differ according their effectiveness in the reduction in computation and memory needed to perform the inference. Most of the techniques lead to a reduction in model size but do not necessarily lead to faster inference without the use of specialized hardware. The chosen works are compared in Table 3.

In the case of pruning, it was observed in [72,74] that the model size can be reduced by more than 50% without significant degradation in STOI, PESQ, and subjectively measured speech intelligibility. Faster inference is possible without specialized hardware when structured pruning is used. However, in the above-mentioned works, it was not tested on actual hardware. Quantization can also lead to a reduction in model size of more than 50%. In some cases (for example, [76]), this at the cost of a small reduction in STOI (by 2.7%). It is also possibly to speed up the quantization when integer computations are appropriately used. For example, in [80], the inference speed was increased by about 20%. In the works about model compression performed with tensor decomposition techniques, high reductions in model size can be achieved. For example, in [93], the size of the parameters was reduced from 17 Mb to 1.216 Mb without a significant reduction in STOI. However, in some cases (for example, [90]), this can be at the cost of slower inference. Pruning and tensor decomposition were directly compared in [93]. It was reported that tensor decomposition gives better STOI than pruning for the same compression rate. KD can lead to reductions in the number of computations (2-4 times), with slight degradation in quality metrics [95]. It was not extensively tested for speech enhancement. Finally, Skip-RNN can reduce the number of operations by 1.78 times [98] without significant degradation in the STOI at the cost of increased memory requirements.

It should be also added that the techniques compared above can be combined to achieve even better speed of inference and lower memory requirements. An example can be found in [72], where pruning was combined with quantization.

### 4.7. Adaptive Computational Load

The variety of hardware limitations on different devices leads to the development of many networks with different memory and computational requirements. There is also a possibility to train one neural network and tune the trade-off between its hardware needs and its performance in terms of quality and intelligiblity metrics. In [52], a method is described in which a neural network is trained such that using it multiple times results in better performance metrics (STOI). Thus, according to the hardware needs, it is possible to decide how many times it should be used. In [100], Bloom-NET was proposed, in which a weak model is defined as a sequence of an encoder, a separator, and mask prediction. The resulting mask is used to multiply the signal from the encoder, and it is then decomposed. In Bloom-NET, the signal from the common encoder is provided at the input of several separators, together with the output from the previous separator. The output of each separator is used to generate a mask, and finally, the encoded signal is multiplied from several masks, and *L* outputs are given and independently decoded.

The decision about the number of layers used to compute the output can also be made during the inference. In [101], the early-exit mechanism (EEM) was proposed. The authors claim that for many input samples, shallow representation is already adequate for classification. In [102], the early-exit mechanism was considered for the speech enhancement task. One aspect of using EEM for speech enhancement is that test examples may vary with SNR. For low SNRs, more layers of the network may be needed to obtain sufficient performance, while for high-SNR examples, shallow networks can be sufficient. The EEM for speech enhancement is implemented by performing a comparison between adjacent stages of the network. If the difference between these outputs is lower than the selected threshold, the model can exit early without passing through all layers. Thus, the system can use less resources when the SNR is high.

## 5. Techniques Used by the Winning Systems in DNS and Clarity Challenges

Speech enhancement challenges provide an opportunity to compare different speech enhancement systems for a specified task. However, it is typically difficult to compare the systems of the participants, as there are many differences between their systems. Yet, it can be very inspiring to see what kinds of systems and which techniques performed the best for the specific task and which techniques seem promising to study. In this section, the results of two challenges are described: the DNS and Clarity Enhancement Challenges. The tasks defined for a given challenge specify different requirements, typically from the perspective of different applications. The target application in the case of DNS is conferencing, while in the case of Clarity, it is speech enhancement for hearing aids. In both challenges, systems are required to be low-latency. In the case of DNS, the processing latency on the specified machine has to be no more than 40 ms, while in the case of Clarity, only algorithmic latency is taken into account, and it cannot exceed 5 ms. The outputs of speech enhancement in DNS are evaluated in terms of speech quality, while in the case of Clarity, the speech intelligibility is the final metric. In DNS and Clarity challenges, both additive noise and reverb are taken into account. The task in the Clarity challenge is, however, not a single-channel enhancement.

### 5.1. DNS INSTERSPEECH-2020

In the first DNS challenge [103], large clean speech and noise datasets were provided. They are 30 times larger than the MS-SNSD challenge described earlier [104]. The test set is divided into four categories with 300 clips in each:

1. Synthetic clips without reverb;
2. Synthetic clips with reverb;
3. Real recordings collected internally at Microsoft;
4. Real recordings from Audioset.

The winning system in the DNS interspeech-2020 challenge was DCCRN (deep complex convolutional recurrent network) [105]. Complex convolutional layers are implemented using regular convolutional layers. For each layer, half of the input features and output activations are treated as real and the other half as imaginary. The convolutions are treated in a way that multiplications and additions are realized as in complex operations. Similarly, batch normalization and LSTMs are also implemented in a way in which multiplications and additions are complex. The structure of the network is the encoder–decoder architecture with two LSTM layers between the encoder and the decoder. Both the encoder and decoder are built using complex convolutions.

Complex batch normalization. The encoder consists of five Conv2D blocks with a kernel and stride of (5, 2) and (2, 1), respectively, and an LSTM of 128/256. Next, the symmetric decoder is built using deconvolution operations. The number of parameters in the DCCRN is about 3.7M. In [105], the DCCRN is compared to the CRN [53], which maps the complex spectrum to the complex mask. However, along with the difference that CRN does not use complex convolutions, the number of layers, kernel sizes, and units are different, and CRN has about twice the number of parameters as DCCRN. Therefore, it is not clear whether the better performance comes from using complex layers or a different size of the network.

### 5.2. DNS ICASSP-2021

In comparison to DNS INTERSPEECH-2020, in DNS ICASSP-2021, twenty hours of clean speech with singing are additionally provided. There is also 100,000 synthetic and real room impulse responses [106] to simulate reverb in synthetic mixtures.

The authors of the winning system in DNS ICASSP-2021 [107] proposed a two-stage complex network with post-processing (TSCN+PP). The idea is based on progressive learning [108]. First, an easier task of mapping a noisy speech magnitude spectrum to a clean speech magnitude spectrum is performed. This task is accomplished with the coarse magnitude estimation network (CME-Net). Next, the resulting estimated magnitude spectrum is combined with the noisy phase to form a coarse complex spectrum (CCS). This estimation is further refined with the complex residual network named the complex spectrum refine network (CSR-Net), which accepts at its input the complex spectrum of the noisy signal and the complex spectrum obtained after the CME-Net. Afterwards, a post-processing network is used for non-natural speech artifacts.

Both CME-Net and CSR-Net have a similar topology, including a gated convolutional encoder, decoder, and stacked temporal convolution modules (TCMs), as proposed in [109]. The gated convolutional encoder uses gated convolutions (using the idea of gated linear units from [110]). The gated convolutional layers in the encoder transform an input spectrogram into a representation with a smaller resolution in frequency dimension and a higher number of channels. The encoder output is processed by the TCM. Finally, the decoder is used to transform the representation obtained from the TCM to the output spectrogram. Finally, post-processing is employed based on the approach from [61]. The idea is to use the gain from the input to the post-processing module as a speech presence probability. This is the basis for obtaining the MMSE-LSA estimator.

### 5.3. DNS INSTERSPEECH-2021

In DNS INTERSPEECH-2021 version [111], both training and test datasets were extended with full-band scenarios. The two tracks in this challenge will focus on real-time denoising for wide-band and full-band scenarios.

The best performing system described in [112] is the speech denoising and dereverberation network (SDD-Net). This is an extended approach from TSCN+PP (see the previous subsection), which contains an additional module for dereverberation. The network consists of a denoising module (DM), a dereverberation module (DR), spectral refinement (SR), and post-processing (PP). Both the DN and DR modules work in the magnitude domain. This is followed by SR, a network that focuses on differences between the output from the previous stages and the target. In DM, DR, and SR, the encoder–decoder structure is used, in which TCMs are applied in the bottleneck. In the PP, a very small network is used as in TSCN. Similarly to TSCN, a multistage paradigm is employed, and before training the network of current stage, the network of the last stage needs to be pre-trained and the freeze weights should be frozen.

In terms of the objective metrics PESQ and ESTOI, the SDD-Net gives the best results after its third stage (SR). However, post-processing additionally improves the DNSMOS metric.

### 5.4. DNS ICASSP-2022

The dataset provided in the DNS ICASSP-2022 [113] is the result of data cleaning and acquisition of more data to capture relevant scenarios. There is a new test set for the full-band condition with new noise types.

The winning system of DNS ICASSP-2022 [114] was a multi-scale temporal frequency convolutional network with axial self-attention (MTFAA-Net) that uses a complex spectrogram at its input and estimates the mask. The network is made up of the following components: a phase encoder, band merging, the main network, band splitting, and masking. The first step is a phase encoder, which maps the spectrum to real-valued features using complex convolutional layers. Next, band merging is performed, which transforms to the ERB scale. This is followed by the main part of the neural network with an encoder–decoder structure, which is built from three types of modules: TF-convolution module, axial self-attention, and frequency downsampling and upsampling. After returning from the ERB frequency scale to the STFT frequencies, masking is performed using a deep filter technique [115]. The ablation study performed by the network authors showed that the removal of the axial self-attention technique has a significant impact on PESQ (0.1), setting the dilations to one, resulting in a further decrease in PESQ (0.16).

### 5.5. Clarity Enhancement Challenge

In the clarity enhancement challenge [116], the task is to improve speech in the context of hearing aids. In contrast to the DNS separation challenge, the main objective was to improve intelligibility instead of quality. It was measured objectively using the MBSTOI metric and objectively using listening tests. However, in this challenge, signals from many microphones were available. The two best teams used Conv-TasNet, which was first developed for single-channel enhancement and later extended to multi-channel conditions.

### 5.6. The Techniques Used in the Winning Systems

The techniques used in the winning systems are shown in Table 4. It can be noticed that in most systems, the complex spectrogram is processed by neural networks. In most of the systems, the encoder–decoder architecture is used. To ensure long-term dependence, TCM is used. However, there are also recurrent layers and self-attention as well. Complex layers (convolutional and recurrent) also turned out to be an effective means of processing complex spectrograms. The results of the comparison of the most systems presented in Table 4 in the same conditions can be found in [112].

## 6. Conclusions and Future Perspectives

In this paper, the aim was to review the techniques and methods used in low-latency DNN-based speech enhancement. Although most of the best-performing systems in DNS challenge work in the spectral domain, the time-domain methods provide good performance and are easy to use in the case of very low-latency requirements (less than 10 ms), which can be concluded from the results of the Clarity challenge.

Many neural networks for the speech enhancement task have an encoder–decoder structure. In their bottleneck, techniques such as recurrent layers, TCMs, or self-attention are used. When the input is in the form of a complex spectrogram, complex layers proved to be effective in encoding the input signal. The mentioned techniques differ in their possibility to set the receptive field, complexity during training, and complexity during testing.

Although many systems are named real-time, their evaluation was not performed on hardware with block-based processing. In order to avoid redundant computations, a complex implementation may be needed.

There are many techniques to compress neural networks. According to an analysis of the literature, such techniques can significantly reduce the requirements for hardware speed and memory. It has also been confirmed in several studies that better performance can be achieved when a large network is trained and then compressed, rather than training small network from scratch. However, compression techniques are rarely used in top-rank systems in DNS and Clarity challenges.

Generally, there is a growing interest in the compression of deep neural networks. This results with new techniques developed also for tasks from other domains (such as language models, image processing). These ideas can be adapted to speech enhancement. Moreover, different neural network compression techniques can be combined to additionally speed-up the inference and reduce the memory requirements.

More efficient deep neural network compression techniques allow for using bigger neural networks that are not only trained with data from diverse acoustic conditions but also networks that use information from other modalities such as video.

As the variety of devices on which DNN-based speech enhancement can be deployed grows, it can be expected that methods that can adapt the requirements of a trained neural network to different hardware limitations will gain significance.

## Figures and Tables

**Table 1:** Summary of DNN-based speech enhancement methods. In size of the dataset, if three values are provided, they mean the number of training, validation, and test utterances, respectively.

Ref.	Inputs	DNN Architecture	Targets	Evaluation Datasets	Size of the Dataset
[7]	Log mel-spectrogram	DNN, RNN	IBM/IRM	TIMIT	-
[8]	Log mel-spectrogram	RNN	IRM	TIMIT	-
[9]	Magnitude spectrogram	DNN, RNN, CNN	Magnitude spectrum	TIMIT + 26 noises from	3696/924/200
[10]	Magnitude spectrogram	Dilated convolutions	IRM/PSM	WSJ0, Auditec, Noisex	320,000
[11]	Magnitude spectrogram	Dilated convolutions	Magnitude spectrogram/IRM	TIMIT, noises, reverberation	34,000
[12]	Windowed time-domain	Dilated convolutions	Windowed time-domain	WSJ0, Auditec, Noisex	320,000
[13]	64-channel cochleagram	LSTM	IRM	WSJ0, Auditec, Noisex	320,000
[14]	Magnitude spectrogram	Convolutional-recurrent	Magnitude spectrogram	-	7500/1500/1500
[15]	Magnitude mel-spectrogram	U-net	Magnitude mel-spectrogram	TIMIT, Noisex	1800/200/192
[16]	Magnitude mel-spectrogram	U-Net with recurrences	Magnitude mel-spectrogram	TIMIT, Noisex	1800/200/192
[17]	Complex spectrogram	U-Net with recurrences, U-net with dilated convolutions	CIRM	WSJ0, TIMIT, Noisex, Freefield	6637/599
[19]	Windowed time-domain	Self-attention	Windowed time-domain	Voice Bank, DEMAND	11,572/824
[20]	Log power spectrogram	U-net with attention	log power spectrum	TIMIT, Noisex	73,920/18,280/200
[21]	Magnitude spectrogram	Convolutional layers, self-attention	IRM/PSM	LibriSpeech, 6 databases with noises	27,538/1000/200

**Table 2:** Comparison of the properties of causal layers for a case when a sequence of *N D*-dimensional vectors is transformed to a sequence of *N H*-dimensional vectors. *K* is the kernel size. $H_{1}$ denotes dimension of query and key vectors in self-attention layers.

Layer	Recurrent	Convolutional	Self-Attention
Receptive field	depends on data	bounded	bounded
Computational complexity	$O(NHD+NH^{2})$	O(NDKH)	$O(NDH+NDH_{1}+N^{2}\left(H_{1}+H\right))$
Maximum path length	O(N)	$O(\log_{K}N)$ (dilated)	O(1)
Sequential operations	O(N)	O(1)	O(1)
Number of the trainable parameters	$O(DH+H^{2})$	O(DKH)	$O(DH_{1}+DH)$
Advantages	no need to set the size of receptive field	fast training and inference	short path between the output and input distant in time
Disadvantages	long training	inference in block-based systems can be complex	computational complexity grows quadratically with context

**Table 3:** Comparison of the properties of the compression methods.

Method	Work	Speed	Model Size	Performance
Pruning	[72]	not tested	67% reduction	no significant degradation in STOI and PESQ
	[74]	not tested	50% reduction	no significant degradation in speech intelligibility
Quantization	[76]	no speedup	50% reduction	reduction in STOI by 2.7%
	[72]			no significant reduction in STOI and PESQ
	[80]	by 20%	71% reduction	better PESQ
Tensor decomposition	[90]	can be decreased	varied	better STOI and PESQ than for pruning for the same compression rate
	[93]	not tested	number parameters from 17 Mb to 1.216	similar STOI and PESQ
KD	[95]	2–4 times lower number of computations		slight degradation in quality metrics
Skip-RNN	[98]	cost of operation reduced 1.78 times	increased memory requirements	not significantly decreased

**Table 4:** Comparison of winning systems in speech enhancement challenges.

Challenge	System	Latency	Time-Dependency	Training Strategy
DNS INTERSPEECH-2020	DCCRN [105]	37.5 ms	Complex convolutions and LSTMs	
DNS ICASSP-2021	TSCN [107]	34.8 ms	TCM	Multi-stage training
DNS INTERSPEECH-2021	SDD-Net [112]		TCM	Multi-stage training
DNS ICASSP-2022	MTFAA-Net [114]	40 ms	dilated convolutions, axial self-attention	
Clarity Enhancement (2021)	Conv-TasNet [117]	5 ms	TCM	

## Data Availability

No new data were created or analyzed in this study. Data sharing is not applicable to this article.
